# Spin-Crossover Molecular Solids Beyond Rigid Crystal Approximation

**DOI:** 10.1186/s11671-016-1398-5

**Published:** 2016-04-14

**Authors:** Iurii V. Gudyma, Victor V. Ivashko

**Affiliations:** Department of General Physics, Chernivtsi National University, Kotsjubynskyi Str. 2, Chernivtsi, 58012 Ukraine

**Keywords:** Spin-crossover nanoparticles, Ising-like model, Phase transition, Hysteresis, Molecular distortion

## Abstract

The qualitative analysis of the spin-crossover molecular solid with distortion effect is presented. A spin-crossover solid with effect of distortion is studied in the framework of the Ising-like model with two-order parameters under statistical approach, where the effect of elastic strain on inter-ion interaction is considered. These considerations lead to examination of the relation between the primary and secondary order parameters during temperature and pressure changes.

## Background

Spin-crossover compounds are a new branch of multifunctional molecule-based magnetic materials. A characteristic feature of these compounds is the molecular spin bistability, where electronic states can be controlled through variation in external stimuli like temperature, light, pressure, magnetic field, chemical species, or a combination thereof [1–3]. A memory effect as a result of bistability is of great interest because this aspect holds the potential for exploitation in memory device units and display of binary data [4–6]. Furthermore, switching between their states makes possible a design of molecular actuators [7], sensing devices [8], thermoelectric nano-devices [9], and cold channel control units in medical storages [10]. Spin-crossover (SC) materials are transition metal complexes with 3*d*^*n*^, where *n*=4…7, electronic configurations which can exist in thermal equilibrium in the high-spin (HS) or low-spin (LS) ground state depending on the strength of octahedral ligand field. For example, the *d*^6^ complexes of ferrous *Fe*^2+^ ion can possess both ground states with total spin *S*=2, ^5^*T*_2_ spectroscopic term (HS) and *S*=0, ^1^*A*_1_ spectroscopic term (LS). In the octahedral symmetry *O*_*h*_, only the HS and LS states are possible [11]. The five *d*-orbitals of bivalent iron ion are split into three *t*_2*g*_− and two *e*_*g*_−orbitals. The spin state is established by the balance between the orbital energy necessary to occupy all accessible 3*d* levels (measured by the size of crystal field *Δ*) and the average energy of the Coulomb repulsion of the *d*-electrons. If these values are approximately equal, the difference between the energy minima of the corresponding HS and LS terms becomes comparable to thermal energy. Under these conditions both states are populated and the system is bistable. In addition, the occupation of the antibonding *e*_*g*_−orbitals in the HS state results in weakening of the iron-nitrogen bonds. Therefore, HS and LS states have dissimilar molecular volumes, and this diversity induces the HS-LS difference in inter-ion distances and generates both short- and long-range interactions [12–17]. It is noticed that the free HS ferrous ion has the larger volume than the LS one.

If the intermolecular interactions are large enough, the thermal transition between the two stable states is associated with a first-order phase transition and is accompanied by a hysteresis loop which is called thermal hysteresis. The inter-conversion between the electronic spin states, occurring in coordination compounds of 3*d* elements, is known as the spin-crossover transition [1–3, 18]. The variation of the fraction of the HS molecules is accompanied by the variation of other thermodynamic parameters and leads to important modifications of the magnetic, optical, and structural properties of material. Therefore, Spiering with co-workers [12] following the models of Slichter and Drickamer [19], and Wajnflasz [20] claimed that the HS fraction *n*_*H*_ of the HS and LS complexes in the crystal is the natural order parameter of the $HS \rightleftharpoons LS$ transition.

If the origin of spin transition phenomenon is essentially molecular, the manifestation of the phenomenon is largely influenced by the cooperativity within the assembly of molecules. The different size of a SC molecule in the HS and LS state leads to cooperativity in this solid [12, 14]. Many experimental and theoretical investigations have implicitly demonstrated that the coupling between electronic states and lattice distortions is a very complex phenomenon and plays an essential role for the high-spin $\rightleftharpoons $ low-spin transition. This gave grounds for Kambara [21] to consider the local (molecular) displacement as the order parameter of the transition. A series of experiments [22, 23] indicates that the direct intermolecular coupling between iron ions can not play any role in the spin state transition but the coupling between ions of iron related to intra- and intermolecular distortions is essential. Recently there are extensive studies of finding a suitable statistical mechanical framework for this features of spin crossover systems [24–26].

Here, we suggest a model that can be used to analyse the combined influence of HS molecules population and lattice distortions on phase transition in spin-crossover solids.

Thus the theory of phase transformations in spin-crossover compounds should be based at least on two order coupled parameters reflecting its magnetic and mechanical nature [27]. The models with two order parameters appear in many problems of condensed matter physics. Moreover, the coupling between magnetic and structural degrees of freedom is one of the sources of the first-order magnetic phase transitions. This is the case, when ferromagnetic exchange interaction depends on the interatomic distance. The second feature of these transitions is the absence of mass transfer, i.e., the phase transition in the spin-crossover materials is diffusionless. It means that the phase transition does not require long-range diffusion during the phase transformation; only slight atomic movements over the distances usually less than the interatomic ones are needed. The atoms maintain their relative relationships during the phase change. The purpose of this research is to give a discussion of the thermodynamic properties of spin-crossover solids with these characteristics.

The paper is organized as follows. First we present the Ising-like model that we use and also analyze the basic equations of developed theory. In the next section, we discuss the results of numerical simulation. In the last section, we summarize the main conclusions.

## Methods

We consider the model of spin-crossover solids with distortion effect in the simplest way. For this, we assume that the deformations are homogeneous and isotropic. In our model, the molecules occupy a simple regular cubic lattice and we restrict their sites to specific points in the regular space. All molecules are arranged equivalently in a solid. The Hamiltonian of the model adopted here is given by

(1) $$ H = -h \sum_{i} s_{i} - \sum_{\substack{ij\\i \not = j}} J_{ij} s_{i}s_{j} + \frac{1}{2}{KV}_{LS}\xi^{2}.   $$

Here, *s*_*i*_ is a fictitious classical spin which has two eigenvalues ±1, corresponding to the LS and HS states respectively, $\sum _{i}$ denotes the sum over all SC molecules. A simple phenomenological approach to the problem is the mean field approximation with the nearest-neighboring interactions in the form $\sum _{j (j \not = i)} J_{ij}\approx zJ$ with coordination number *z* (the number of the first neighbours of a given molecule in the lattice). Variable *ξ* indicates the change of the relative inter-ion distance and for *ξ*=0 we have the rigid lattice, *V*_*LS*_ is the volume of the molecule in the low-spin state. In order to describe and understand the behavior of isotropic elastic media with classical spins, based on a chemical/physical intuition, similar Hamiltonian has often been considered as phenomenological one.

In the present model, the effects of ligand molecules on the transitions are taken into account mainly through the energy separation *h* between the ^1^*A*_1*g*_ and ^5^*T*_2*g*_ states. On the discrete level approach, for the isolated magnetic molecule, the uniform “magnetic field” *h* is generally the energy distance between the HS and the LS states

(2) $$ h = - (\Delta - k_{B} T \ln g + p \delta V_{LH}),   $$

where the parameter *Δ* is directly related to the strength of crystal field per site at ambient pressure, *k*_*B*_*T* is the thermal energy, *g*=*g*_*H*_/*g*_*L*_ is the electrovibrational degeneracy ratio between the HS and LS states of SC molecule. Traditionally, we simply take *g* as a constant. The last term takes into account the applied constant pressure. Here, *p* indicates the external uniform pressure, *δV*_*LH*_ is the change in molecular volume during the spin crossover.

The HS fraction *n*_*H*_ is the fraction of molecules occupying the HS state, and it is the natural order parameter of the $HS\rightleftharpoons LS$ transition. Since *n*_*H*_+*n*_*L*_=1 and *n*_*H*_−*n*_*L*_=<*s*>, $n_{H}=\frac {1}{2}(1+\left <s\right >)$. Thus, we can obtain $n_{H}=n_{L}=\frac {1}{2}$ for the equilibrium temperature *T*_*eq*_. The dimensionless “fictitious magnetization” per site $\left <s\right >\equiv N^{-1} \sum _{i} s_{i}$ has been constructed to take values +1(−1), angular brackets denote average of ensemble. Here *N* is number of transition metal ions.

The second term in the Hamiltonian (1) describes the intermolecular interactions of elastic origin through a phenomenological parameter accounting the ferromagnetic coupling (*J*>0) between neighboring spins *i* and *j* in Ising form. This is the simplest way to express the cooperativity between magnetic molecules [28–34]. Thus in the first approach we merely took into account the existence of cooperativity without giving details of its origin. The intermolecular interaction *J* is given by some short-range potential whose explicit form is not considered in the paper but these interactions are always present in real systems.

The third term is the elastic energy of the distortion lattice, which is represented in the harmonic approximation. The zeroth level of elastic energy is counted from thermal equilibrium state wherein fractions of molecules in various states are the same. We consider that all the molecules in a given spin state are characterized by only one effective mean harmonic strain. We quantify the distortion by one geometrical parameter, which is the symmetrized strain *ξ*. This is a common and sufficient approximation to describe spin crossover phenomena. Here, *K* is the bulk modulus of the solid lattice (the volumetric elastic constant). We have assumed the relaxed (<*s*>=0) state at any temperature as unstrained, thus we have neglected the thermal expansion. Moreover, in this simplest consideration, we ignore a possible anisotropy of the solid.

So far, the effect of coupling between the fictitious spin (spin state of molecule) and the distortion has been neglected. This coupling gives rise to spontaneous strains in the process of ordering of the quasi-spins. As far as during the spin crossover transition, in most cases, there is no change in crystal symmetry, the spontaneous all-round full symmetric lattice distortion is supposed to arise from the spin interconversion. A more realistic treatment, which includes the misfit between the HS and LS molecular volumes leads to strong elastic interactions, which play a major role in the cooperativity of spin transition. We will take into account the major origin of the relationship between a cooperativity and crystal lattice in a spin-crossover molecular solids as a simple series expansion *J* upon homogeneous elastic strain which occurs for small compressibility

(3) $$ J = J_{0} + J_{1} \xi + J_{2} \xi^{2}.   $$

Here, *J*_0_ is the “exchange” function from the rigid-lattice value, *J*_1_ and *J*_2_ is the first and second of the exchange integral by the distortion. This form of inter-site interaction corresponds to an indirect effect of distortion on temperature spin transitions. The external pressure effect on intermolecular interaction will contribute through the distortion *ξ*. As mentioned above the case *ξ*=0 corresponds to the rigid crystal approximation.

By neglecting fluctuations on a semi-macroscopic level of description in the nearest neighbor approach the functional of free energy per molecule has been derived analytically

(4) $$ g = \frac{G}{N} = z J \left<s\right>^{2} + \frac{1}{2} K V_{LS} \xi^{2} - k_{B}T \ln \left[2\cosh (x)\right],   $$

where *x*=(2*zJ*<*s*>+*h*)/*k*_*B*_*T*, <*s*>=2*n*_*H*_−1. Hereinafter, *K*=*K*/*N*.

Minimization of the free energy with respect to *n*_*H*_ and *ξ* leads to the coupled self-consistent equations, i.e., the equations of state in thermodynamic equilibrium,

(5) $$\begin{array}{@{}rcl@{}} &n_{H} = \frac{1}{2}\left[1+\tanh\left(x \right)\right],  \end{array} $$

(6) $$\begin{array}{@{}rcl@{}} &\xi = \frac{z J_{1} \left<s\right>^{2}}{K V_{LS} - 2 z J_{2} \left<s\right>^{2}}.  \end{array} $$

The obtained self-consistent equations for the mean HS fraction *n*_*H*_ and distortion *ξ* have a set of non-trivial solutions determined by different relations between the characteristic parameters of short- and long-range interactions. External pressure also can significantly affect the type and the temperature of spin crossover transition. The physical solutions of this set of coupled equations minimize the free energy and define the equilibrium properties of the system. Values *n*_*H*_ and *ξ* are primary and secondary coupled order parameters. Thus, in the study of spin-crossover transition, two different quantities may serve as order parameters. The primary order parameter quantifies the path along the phase transition, while secondary order parameter describes other modes of the elastic response of the material. It should be noticed that the lattice changes are reflected in the sum of strain caused by the magnetization and the applied external pressure. From high temperature expansion of the function in Eq. (5), we have the following ${T_{c}^{0}}$:

(7) $$ {T_{c}^{0}} = \frac{2 z J_{0}}{k_{B}}.   $$

Now, if the temperature ${T_{c}^{0}}$ is replaced by *T*_*eq*_, the critical interaction parameter ${J_{0}^{c}}$ may be defined as

(8) $$ {J_{0}^{c}} = \frac{k_{B} T_{eq}}{2 z}.   $$

Next, we will show that the values $T_{eq},{J_{0}^{c}}$ determine a critical point. If *J*_0_=*J*_1_=0, we reproduce, naturally, the case of SC theory traditionally studied [20].

## Results and Discussion

**Fig. 1:**
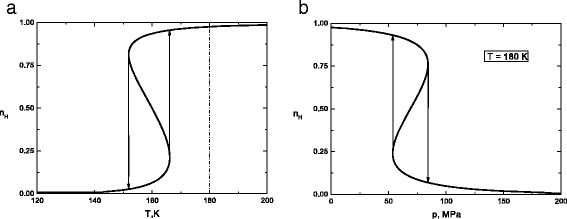
Variation of the HS fraction *n*_*H*_ of molecules with temperature *T* (**a**) and pressure *p* (**b**). The *left-hand* panel corresponds to the pressure absence situation

Due to the strong non-linear character of the functions *n*_*H*_, *ξ*, Eqs. (5), (6) have to be solved numerically. The corresponding order parameters are plotted in Fig. 1 as the functions of temperature *T* and pressure *p*.

Since the coordination number for 2D system is *z*=4, we set the parameter ${J_{0}^{c}}=30 K$ that gives a realistic value for the transition temperature ${T_{c}^{0}}=240~K$. In our simulations, the following typical values for SC materials featuring a spin transition were used: *Δ*=800*K*, ln*g*=5. The other values of parameters used in present calculations are *K*=2.15·10^6^*N*/*m*^2^, *V*_*LS*_=2400Å^3^, *δV*_*LH*_=20Å^3^, *J*_1_=10*K*, *k*_*B*_=1.

For these parameters the system exhibits an abrupt thermal spin transition centered around 160 K without external pressure that corresponds to equilibrium temperature. Generally, the thermal behavior of the dynamic order parameters characterizes the nature of the phase transitions that may be discontinuous or continuous, depending on the choice of the values of *Δ*, *p*, *J*_0_, *J*_1_, and *J*_2_. In our calculation of the parameter *J*_2_ is taken to be zero because it has little effect on the behavior of system. It must be noticed that the transition from the nonmagnetic phase to the paramagnetic phase and vice versa is accompanied by a little structural change. The averaged distance between iron and surrounding nitrogen atoms depends on the spin state. External pressure can change the distance, and hence leads to a change in the spin state of the magnetic ion. As previously noted, pressure favors the smaller LS complexes, thus it stabilizes the LS phase of compound [35]. Therefore, experimental and theoretical studies of spin crossover compounds concern the influence of the pressure on the thermal hysteresis are important. It is well known that by applying a pressure on SC, one modifies linearly “magnetic field” *h* according to the Eq. (2). This translates into a linear increase of the experimental switching temperatures *T*_*↑*_ and *T*_*↓*_ with applied hydrostatic pressure and reduction of the hysteresis width down to zero [36, 37]. Usual *T*_*↑*_ and *T*_*↓*_ are the transition temperatures on heating and cooling at *n*_*H*_=0.5.

In Fig. 1b, pressure-induced transition which is presented for temperature *T*=180 *K* for which the thermal spin transition has already been occurred as it is seen in the left-hand panel. It is evident that the transition pressure of the HS-LS transition is greatly influenced by choice of the parameter ${J_{0}^{c}}$ and temperature *T*. This model can reproduce gradual or abrupt pressure induced transitions, with or without pressure hysteresis, by tuning the values of the model parameters. If the calculations are followed for an increasing pressure sequence at the temperature *T*=180 K, the transition is found to be centered at *p*_*↓*_=84.3 MPa, whereas the transition pressure for decreasing pressure calculations is significantly lower, being centered at *p*_*↑*_=53.6 MPa. The equilibrium value of pressure for which $n_{H}=n_{L}=\frac {1}{2}$ is *p*_*eq*_=69 MPa.

By increasing the applied pressure, we notice that the thermal hysteresis loop of HS fraction becomes more narrow up close. A similar effect is observed in the pressure dependence of the HS fraction which is considered under increasing temperature. The equilibrium temperature *T*_*eq*_ and pressure *p*_*eq*_, respectively, are shifted towards higher values. It must be noticed that the equilibrium temperature and pressure formally determine the points at which Hund’s rule on electronic ground state of transition-metal ion breaks down. The expected behavior of SC solids under pressure is many times observed on experiments [1]. Also it must be noticed that the constants *J*_1_ and *J*_2_ determine the asymmetry of transition curves. The larger these parameters are chosen, the more transition curves are asymmetric.

**Fig. 2:**
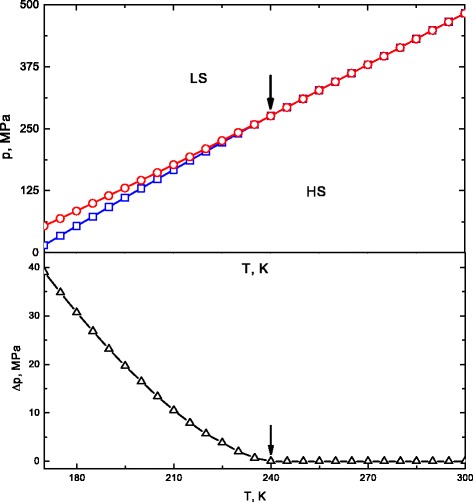
(*Color online*) Temperature dependence of the spin transition pressures *p*_*↑*_ (*blue line*) and *p*_*↓*_ (*red line*) (*top panel*) and pressure induced hysteresis width (*bottom panel*) at HS fractions *n*_*H*_=0.5

Due to the fact that SC materials are both thermal and pressure sensitive, it is very important to analyze the pressure-temperature (*p*−*T*) phase diagram. Furthermore, the temperature and external pressure are two independent variables for which a phase diagram with LS and HS regions can be constructed. We have designed diagram of SC system and its pressure-induced hysteresis width versus temperature. The calculated results are reported in Fig. 2, and they exhibit the effect of temperature on the pressure induced hysteresis of the spin transition.

Up to critical temperature ${T_{c}^{0}}=240~K$ indicated by arrows the width of pressure hysteresis is narrowed with increased temperature and vanishes in transition point. It is evident that above the critical temperature the transition occurs gradually. In this case, the effect of temperature is appreciable and manifests itself not only in the change of the transition pressure but also in the change of the type of the spin-crossover transition. The LS phase was found to be stable at high pressure above the red line *p*_*↓*_. Conversely HS phase is stable at lower pressures below the blue line *p*_*↑*_. There is an intermediate bistable region of coexistence of both phases. From theoretical point of view, the phase diagram gives insight into the factors contributing to transition. As can be seen from phase diagram in the top panel of Fig. 2, the transition from the HS to the LS state can be achieved either by decreasing the temperature or by increasing the pressure.

**Fig. 3:**
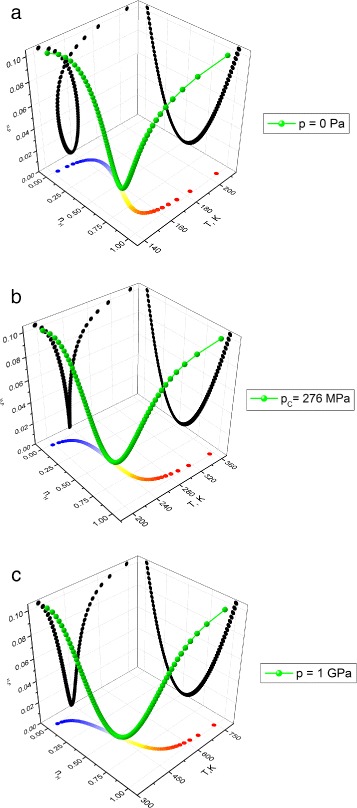
(*Color online*) The distortion *ξ* as a function of temperature *T* and HS fraction *n*_*H*_ and its projections on coordination planes for different values of pressure *p*. The pressure is increasing as is shown on the right at each figure. The 3D curve (*green online*) corresponds to the distortion; on the bottom projection *n*_*H*_−*T* the *blue dots* correspond to the low-spin state and the *red dots* correspond to the high-spin state

Bearing in mind the fact that the spin-crossover transition is accompanied concomitant molecular change, comparison between the characteristic 3D maps of distortion *ξ* as a function of temperature *T* and HS fraction *n*_*H*_ for different values of pressure *p* was calculated. Based on our parameters, the corresponding 3D plots are summarized in Fig. 3.

One can easily remark that a curve distortion is twisted on temperature in the region of existence of thermal hysteresis loops. The increasing of pressure untwists the curve. Figure 3 shows a distortion transfer from hysteresis transition to gradual transition of spin-crossover system which is especially clear from the projections in the plan *n*_*H*_−*T*. Such a fact corresponds to the usual pressure effect in SC solids that is accompanied by the vanishing of the first-order character of the transition at high-pressure values. Note that the hysteresis of the spin state corresponds to the thermal butterfly loops of distortion. With increasing pressure *p* the evolution of the associated thermal butterfly loops width shows also an decreasing behavior. The behavior of a distortion-like secondary order parameter displays principal features of transition in spin-crossover solids. The distortion reaches a minimum at the point of equilibrium where fractions $n_{H}=n_{L}=\frac {1}{2}$ that is the consequence of its approximation of symmetrization adopted in the previous section.

**Fig. 4:**
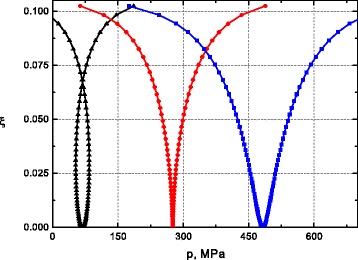
(*Color online*) Functional dependence the distortion *ξ* on pressure *p* for several temperatures going from 180 K (*black triangles*) to 300 K (*blue squares*) (*left to right*). In the middle, the dependence (*red circles*) corresponds with the critical pressure *p*_*c*_=276 *M* *P* *a* and the critical temperature ${T_{c}^{0}}=240~K$

From the results shown, it is of interest to extract the functional relationship between the distortion *ξ* and the external pressure *p* for various temperatures. Numerical solutions for Eq. (6) for different *T* are illustrated in Fig. 4 typical pictures as function *p*.

We have defined “fictious magnetization” <*s*> from associated Eq. (5). To the critical pressure *p*_*c*_=276 *MPa*, the distortion takes the form of butterfly loops. For the critical pressure *p*_*c*_=276 *MPa* and the critical temperature ${T_{c}^{0}}=240~K$, the butterfly loops collapses and transfers to a V-shaped curve for further increasing of pressure. These and previous results underline the importance of the inclusion of the distortion contribution like secondary order parameter. Research of joint change in the high-spin fraction of molecules and the distortion give the full picture of what is happening in the spin-crossover solid system. The resulting form of the distortion corresponds to made earlier approximation of the symmetrized strain (see, section Methods). We may conclude that assuming of the symmetrized strain the equilibrium occurs naturally at identical quantity of molecules in the high-and low-spin states. If equilibrium is disturbed, the distortion becomes different from zero, but it remains symmetrical. As one would expect, the equilibrium point is shifted to higher values of pressure when the temperature increases as illustrated in Fig. 4. This explanation remains valid as soon as we neglect the anharmonic effects that enhance the thermal dependence of the molecular volume change.

The critical curve that separates the hysteretic behavior from the non-hysteretic one is known as spinodal curve. Starting from Eq. (5), we obtain the dependence of temperature *T* as a function of “fictious magnetization” <*s*>:

(9) $$ T = \frac{2 z J <s>- \Delta - p \delta V_{LH}}{k_{B} (\arctan(h <s>)-\ln (g))}.   $$

Next, the spinodal curve can be found by computing minimum *dT*/*d*<*s*>=0. Thus, the spinodal curve will be described by the following expression:

(10) $$\begin{array}{*{20}l} & \frac {dT}{d<s>}=\frac{2 z J}{\arctan(<s>)-\ln (g)} \notag\\ & - \frac{2 z J <s>- \Delta - p \delta V_{LH}}{(1 - <s>^{2}) (\arctan(<s>)-\ln (g))^{2}}=0.  \end{array} $$

**Fig. 5:**
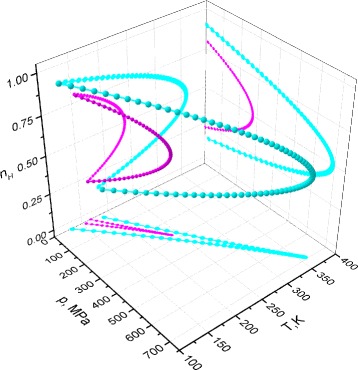
(*Color online*) *p*−*T*−*n*_*H*_ spinodal curves and its projections on coordination planes. Curves obtained for the interaction parameters *J*_0_=30 *K* (*magenta*) and *J*_0_=45 *K* (*cyan*) for comparison

Figure 5 represents some spinodal curves and its projections on coordination planes *n*_*H*_−*T*, *n*_*H*_−*p* and *p*−*T* for different values of intermolecular interaction parameters *J*_0_.

The difference in the stability domains of the phases in the various cases can be qualitatively understood as follows. For each system, there is a critical temperature *T*_*c*_, above which hydrostatic pressure does not induce the HS–LS phase transition anymore. Indeed, the difference in critical temperatures can be accounted for by a larger interaction energy between molecules.

On other hand, the equilibrium temperature *T*_*eq*_ is obtained by setting <*s*>=0 in Eq. (9). Thus, we have the mean-field approach depending of the equilibrium temperature *T*_*eq*_ on the external pressure *p*

(11) $$ T_{eq} = \frac{\Delta + p \delta V_{LH}}{k_{B} \ln (g)}.   $$

Obviously, here, *T*_*eq*_ is an increasing function of pressure because the low-spin state has a smaller volume.

The above considerations are very general and obviously not limited to the special case of one or the other SC crystalline solids.

## Conclusions

In this paper, we have presented a self-consistent mean-field theory for a lattice-spin model of SC solids that exhibits a first-order phase transition. The essential features of the model are (1) the system can be regarded as a set of subsystems of the lattice and two-level spins located in each site and (2) molecular distortion is included indirectly in the intermolecular interactions. The second feature plays an important role in the physics of SC solids inasmuch as lead to two parameters of order: the HS fraction of molecule-like primary parameter and the molecular distortion-like secondary parameter.

For the problem discussed here, calculations are made in a model of isotropic elastic continuum generally used in treating LS-HS transitions. In real crystals, however, the elastic interactions are more complicated. This gives rise to a more complex problem which will be discussed in a forthcoming work.

Taking into account the solid strain, a simple Ising-like model with applied pressure was considered. On the basis of this model the free energy per spin-crossover molecule that makes possible to derive state equations of the system was obtained. The kind of phase transition in general depends on the magnitude of inter-ion interaction *J*_0_. The results demonstrate that the increase of the strain component leads to the forthcoming first-order phase transition. The obtained phase diagram gives a more complete description of diffusionless processes occurring in the spin-crossover system.
